# Phosphodiesterase type-5 inhibitor use in type 2 diabetes is associated with a reduction in all-cause mortality

**DOI:** 10.1136/heartjnl-2015-309223

**Published:** 2016-08-01

**Authors:** Simon G Anderson, David C Hutchings, Mark Woodward, Kazem Rahimi, Martin K Rutter, Mike Kirby, Geoff Hackett, Andrew W Trafford, Adrian H Heald

**Affiliations:** 1 Institute of Cardiovascular Sciences, University of Manchester, Manchester, UK; 2 The George Institute for Global Health, Oxford Martin School, University of Oxford, Oxford, UK; 3 The George Institute for Global Health, University of Sydney, Australia; 4 Manchester Diabetes Centre, Central Manchester University Hospitals NHS Foundation Trust, Manchester, UK; 5 Centre for Endocrinology and Diabetes, Institute of Human Development, University of Manchester, Manchester, UK; 6 The Centre for Research in Primary & Community Care, University of Hertfordshire, Hatfield, UK; 7 Department of Urology, Good Hope Hospital, Sutton Coldfield, UK; 8 School of Medicine, and Manchester Academic Health Sciences Centre, University of Manchester, Manchester, UK; 9 Leighton Hospital, Crewe, UK

## Abstract

**Objective:**

Experimental evidence has shown potential cardioprotective actions of phosphodiesterase type-5 inhibitors (PDE5is). We investigated whether PDE5i use in patients with type 2 diabetes, with high-attendant cardiovascular risk, was associated with altered mortality in a retrospective cohort study.

**Research design and methods:**

Between January 2007 and May 2015, 5956 men aged 40–89 years diagnosed with type 2 diabetes before 2007 were identified from anonymised electronic health records of 42 general practices in Cheshire, UK, and were followed for 7.5 years. HRs from multivariable survival (accelerated failure time, Weibull) models were used to describe the association between on-demand PDE5i use and all-cause mortality.

10.1136/heartjnl-2015-309223.supp1 Supplementary appendix

**Results:**

Compared with non-users, men who are prescribed PDE5is (n=1359) experienced lower percentage of deaths during follow-up (19.1% vs 23.8%) and lower risk of all-cause mortality (unadjusted HR=0.69 (95% CI: 0.64 to 0.79); p<0.001)). The reduction in risk of mortality (HR=0.54 (0.36 to 0.80); p=0.002) remained after adjusting for age, estimated glomerular filtration rate, smoking status, prior cerebrovascular accident (CVA) hypertension, prior myocardial infarction (MI), systolic blood pressure, use of statin, metformin, aspirin and β-blocker medication. PDE5i users had lower rates of incident MI (incidence rate ratio (0.62 (0.49 to 0.80), p<0.0001) with lower mortality (25.7% vs 40.1% deaths; age-adjusted HR=0.60 (0.54 to 0.69); p=0.001) compared with non-users within this subgroup.

**Conclusion:**

In a population of men with type 2 diabetes, use of PDE5is was associated with lower risk of overall mortality and mortality in those with a history of acute MI.

Erectile dysfunction (ED) is increasingly recognised as an early marker of cardiovascular disease (CVD).^1^ The association of ED with CVD increases in the presence of type 2 diabetes,^2^ ^3^ and up to a third of those with ED have a comorbid condition including hypertension,^4^ peripheral vascular disease (PVD), obesity,^5^ smoking or dyslipidaemia.^6^ ^7^ Phosphodiesterase type-5 inhibitors (PDE5is) are considered the first-line therapy for treatment of ED^8^ and, since their introduction in 1998, concern for their use has diminished in high-risk patients with CVD.

In fact, PDE5is have been shown to confer systemic vascular benefits against the development of major adverse cardiac events.^9^ This is supported by compelling evidence from animal models indicating that the PDE5is such as sildenafil, tadalafil and vardenafil are cardioprotective in ischaemia-reperfusion injury (IRI) while suppressing cardiac arrhythmias and improving cardiac function.^10–15^ Furthermore, small randomised controlled trials (RCTs) in systolic heart failure have shown an improvement in haemodynamic parameters and functional indices.^16^ It is therefore important to establish whether there is benefit or harm in high-risk patients with ED.

Over the past decade, PDE5is have attained a proven safety profile, with no evidence of harm due to acute myocardial infarction (AMI) or sudden cardiac death,^17^ and data from clinical trials indicate a relatively low incidence of cardiovascular adverse events compared with placebo-treated patients, with no increase in cardiac mortality compared with mortality rates in age-standardised male populations.^18^ ^19^ There are, however, no definitive long-term prospective data on the impact of PDE5is on mortality. Meta-analyses of randomised, placebo-controlled trials to evaluate the efficacy^20^ and safety of PDE5is on cardiac function suggested that PDE5i use contributes independently to improved cardiac function with good safety profiles.^21^

In a population-based study, we sought to determine whether use of PDE5is in patients with type 2 diabetes mellitus and ED was associated with a reduction in 7-year mortality. The primary hypothesis of this observational primary care-based study was that men with type 2 diabetes ever treated with a PDE5i would have better overall survival than those who were never treated.

## Methods

### Data source

We examined pseudo-anonymised electronic health records in a retrospective cohort of men aged 40–90 years, known to have type 2 diabetes attending 42 general practices (GPs) in Central and Eastern Cheshire, UK. Individuals were eligible for inclusion if they had diagnosis of diabetes prior to the cohort entry date of 1 January 2007 to allow follow-up of at least 5 years. A retrospective cohort was identified from the electronic health records using relevant Read clinical codes for type 2 diabetes. Read codes are a hierarchical clinical coding system of over 80 000 terms that are used in GPs in the UK.^22^ Data were searched by Egton Medical Information Systems (EMIS), a commercial organisation that provides health information systems for the majority of family practices in Cheshire.

### Exposure

Of the total cohort, we identified men who had received a prescription of a PDE5i (sildenafil, vardenafil or tadalafil) prior to 1 January 2007 and at least once, up to 30 June 2015. As PDE5is are prescribed to be taken *pro re nata* and not continuously, the mean length of treatment with a PDE5i could not be accurately established for these analyses. We identified the number of times PDE5is was prescribed from the electronic health records. We have no information regarding frequency of use once a prescription has been filled.

### Variables

We collected data on potential confounders, including age, blood pressure (BP), weight, height (and body mass index (BMI) derived from weight and height), HbA1c, creatinine, glucose, total cholesterol, high-density lipoprotein (HDL) and low-density lipoprotein (LDL) cholesterol and triglycerides, during 2007 and used the mean values of multiple measurements, where available, in our analyses. Assays were performed in the Departments of Biochemistry at Macclesfield and Leighton Hospitals, Cheshire, UK. HbA1c, creatinine, glucose and lipids were analysed on the Vitros 5.1 autoanalyser (Johnson & Johnson, Rochester, New York, USA). We obtained information on other cardiometabolic risk factors including a history of stroke, transient ischaemic attack (TIA), previous MI, PVD, congestive cardiac failure and atrial fibrillation from the primary care pseudo-anonymised records.

### Reference population

A reference cohort of men (aged 40–89 years), without a prior history of diabetes or PDE5i use as of 1 January 2007 to 30 June 2015 was also obtained by random selection, from a search by EMIS, of the same GP records.

### Outcomes

For all patients, the main outcome was death from any cause during the study period. Currently, the reporting of deaths within primary care is well established, and if a patient dies in secondary or tertiary care, a GP must be notified of the death. We ascertained the date of deaths from GP records. We also examined the electronic health records to identify prior history (before or on 1 January 2007) of a MI using relevant Read codes for MI. This allowed the determination of secondary outcome—incident AMI, defined as events occurring after 1 January 2007. We controlled for survival bias by following all participants from the same point in time—a landmark of 6 months post 1 January 2007. Follow-up was censored at death, on the date an individual left the practice or at the last data collection for the practice (30 June 2015), whichever occurred first.

### Statistical analysis

All analyses were conducted using the Stata V.13.1 (College Station, Texas, USA). Categorical variables were compared using χ^2^ test or Fisher's exact test, continuous variables were compared using t tests or analysis of variance, and Kaplan-Meier curves were used to summarise survival. Kaplan-Meier curves were fitted to compare survival probabilities for men with no history of type 2 diabetes or PDE5i use and those with type 2 diabetes, stratified by PDE5i use. We investigated proportional hazards assumption by tests and graphical diagnostics based on scaled Schoenfeld residuals. A test of the proportional hazards assumption was obtained by correlating the corresponding set of scaled Schoenfeld residuals with the Kaplan-Meier estimate of the survival distribution.

We fitted Poisson regressions from the occurrence (count) of an incident MI (per person) during follow-up and reported unadjusted and age-adjusted incidence rate ratios (IRRs) from the transformed coefficients using the *Poisson* command in Stata. An assessment for equidispersion (data not shown) was performed using post-estimation tests, following Poisson regression, for deviance goodness-of-fit and Pearson goodness-of-fit.

A time-dependent variable for PDE5i initiation was used to define users and non-users of PDE5is. Follow-up started from a landmark of 6 months post 1 January 2007 until death or the end of follow-up period. For PDE5i users, the value of the time-dependent variable is 0 before the time of first prescription and changes to 1 thereafter when a PDE5i is prescribed. For the non-users, the value remains as 0 in the follow-up period. This method has been shown to accurately represent the exposure status and classifies the ‘event-free’ person-time of the users before their first prescriptions as the unexposed follow-up time.^23^ ^24^

We developed a multivariable survival (accelerated failure time, Weibull) model to estimate HRs for mortality in those who were and those who were not prescribed a PDE5i (included as a time-dependent variable), adjusting for age category, smoking status, modification of diet in renal disease (MDRD) defined estimated glomerular filtration rate (eGFR), a history of cerebrovascular disease (CVD), MI, hypertension and history of statin, aspirin, β-blocker and metformin prescription. Incident MI was similarly analysed.

In the subgroup of men (n=432) who had an incident MI during follow-up, the time to censor began from the reported date of MI to the date of death, from the date an individual left the practice or from the last data collection for the practice (30 June 2015), whichever occurred first.

Sensitivity analyses to estimate similarly adjusted multivariable survival (accelerated failure time, Weibull) models were determined. The first sensitivity analysis used multiple imputations with chained equations (linear regression for continuous and logit regression for categorical variables) to impute missing data (eGFR, triglycerides, HbA1c, total cholesterol, HDL cholesterol, smoking category, Townsend score, systolic and diastolic BP). The Nelson-Aalen estimate as well as time at risk was included as covariates. Twenty imputations were generated. The second sensitivity analysis used propensity score matching to further account for confounding, selection and prescription bias using these observational retrospective data. Mahalanobis nearest-neighbour matching was performed on the imputed cohort within a calliper of 0.2 standard deviation of the logit function of propensity scores to create (1:n) matched groups (see online supplementary table S1). The third sensitivity analysis used a Fine-Gray regression model (Stata module *stcrreg*) for competing risks,^25^ on both all-cause mortality and competing incident MI as a function of the same covariates (see online supplementary figure S1). This is because of the possibility that death and an incident MI might confound each other.

## Results

A total of 5956 men with known type 2 diabetes aged 40–89 years were identified at cohort entry date of 1 January 2007. Of the total, 1359 (22.8%) received a PDE5i, prescribed a median 16 times (IQR 2–173). The baseline characteristics of these individuals are shown in table 1. Men who were prescribed a PDE5i for ED were slightly younger at 71.3 years (95% CI 70.6 to 71.7) versus 72.8 years (72.4 to 73.1), had a slightly higher BMI and a lower rate of current smoking (22.9% vs 25.1%; table 1). Aspirin use was greater in PDE5i users but no difference was noted in the proportion of men who were prescribed clopidogrel. Mean systolic and diastolic BP levels were similar, with more than two-thirds of the population having a history of hypertension (71% in users vs 67% in non-users). The eGFR levels in the PDE5i group were significantly higher but serum cholesterol and LDL were similar. A greater proportion of men treated for ED were prescribed a statin.

**Table 1:** The demographic and clinical characteristics at baseline of men with type 2 diabetes in the study population

	Not prescribed a PDE5i	Prescribed a PDE5i
N (%) or mean (95% CI)	4597 (77.2)	1359 (22.8)
Median number of prescriptions†		16 (2–173)
Age (years)	72.8 (72.5–73.1)	71.2 (70.6–71.7)**
HbA1c, %	7.3 (7.2–7.3)	7.4 (7.3–7.5)*
BMI (kg/m^2)^	30.3 (30.1–30.6)	30.6 (30.1–31.0)
LDL (mmol/L)	2.21 (2.18–2.23)	2.21 (2.16–2.25)
HDL (mmol/L)	1.16 (1.14–1.17)	1.16 (1.14–1.18)
Cholesterol (mmol/L)	4.08 (4.05–4.11)	4.10 (4.05–4.16)
Albumin g/L	41.6 (41.5–41.7)	41.5 (41.3–41.6)
Creatinine μmol/L	104 (102–105)	100 (98–102)*
eGFR (MDRD)	68 (67–69)	70 (69–71)**
Systolic BP (mm Hg)	138 (137–138)	138 (137–139)
Diastolic BP (mm Hg)	77 (76–77)	77 (76–77)
Smoking status		
Never smoked	1326 (30.0)	408 (30.8)
Ex-smoker	1987 (44.9)	614 (46.3)
Current smoker	1113 (25.1)	304 (22.9)
Any statin use	2997 (65.2)	1092 (80.4)**
Clopidogrel use	237 (5.2)	55 (4.1)
Aspirin use	1894 (41.2)	714 (52.4)**
Metformin use	2126 (46.3)	874 (64.3)**
β-blocker use	1303 (28.3)	338 (24.9)*
History of prior MI	840 (18.3)	191 (14.1)**
History of atrial fibrillation	680 (15.0)	165 (12.4)*
Hypertension	3062 (66.6)	963 (70.9)**
Peripheral vascular disease	518 (11.3)	145 (10.7)
Stroke	367 (7.8)	65 (5.0)**
TIA	325 (7.1)	78 (5.7)
Congestive cardiac failure	511 (11.1)	108 (8.0)**

Data are most recent measure within 1 year of study entry.

**p<0.001; *p<0.05.

†Median and IQR.

BMI, body mass index; BP, blood pressure; eGFR, estimated glomerular filtration rate; HDL, high-density lipoprotein; LDL, low-density lipoprotein; MI, myocardial infarction; PDE5i, phosphodiesterase type-5 inhibitor; TIA, transient ischaemic attack.

### 

#### All-cause mortality

**Figure 1:**
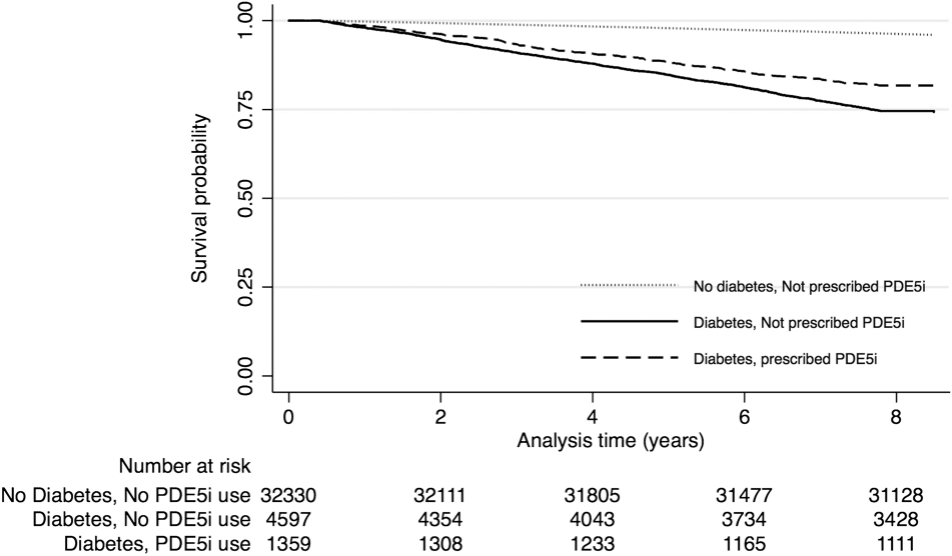
Time to all-cause mortality Kaplan-Meier curves by treatment (PDE5i vs no PDE5i) groups with history of diabetes and including non-users with no history of diabetes (reference population). PDE5i, phosphodiesterase type-5 inhibitor.

Participants were followed up for a mean of 7.5 years (range 0.4–8.5 years). From the same background population, a random (reference) sample of 32 330 men without diabetes or PDE5i use, followed up for a mean 8.3 years, had mortality rates of 4.8 (4.5 to 5.0) per 100 person-years. Fewer deaths (age adjusted) occurred among PDE5i users with type 2 diabetes: 248 (19.1%) versus non-users (1170, (23.8%); 2=12.47, p=0.0004). The mean follow-up time was 7.7 years (95% CI 7.6 to 7.7) versus 7.4 years (7.3 to 7.5) in the PDE5i user and never-prescribed groups, respectively. Similarly, all-cause mortality rates (per 1000 person-years) were lower in PDE5i-treated patients (25.2 (22.3 to 28.5) vs 34.0 (32.9 to 36.9); p<0.0001). Men on treatment with a PDE5i had a 31% lower risk of all-cause mortality compared with non-users (figure 1; HR=0.69 (0.60 to 0.79); p<0.001). In a sensitivity analysis of a propensity score-matched cohort of 621 men (see online supplementary table S1), the estimated unadjusted hazard for mortality associated with PDE5i use was 0.64 (0.44 to 0.92; p=0.017).

**Figure 2:**
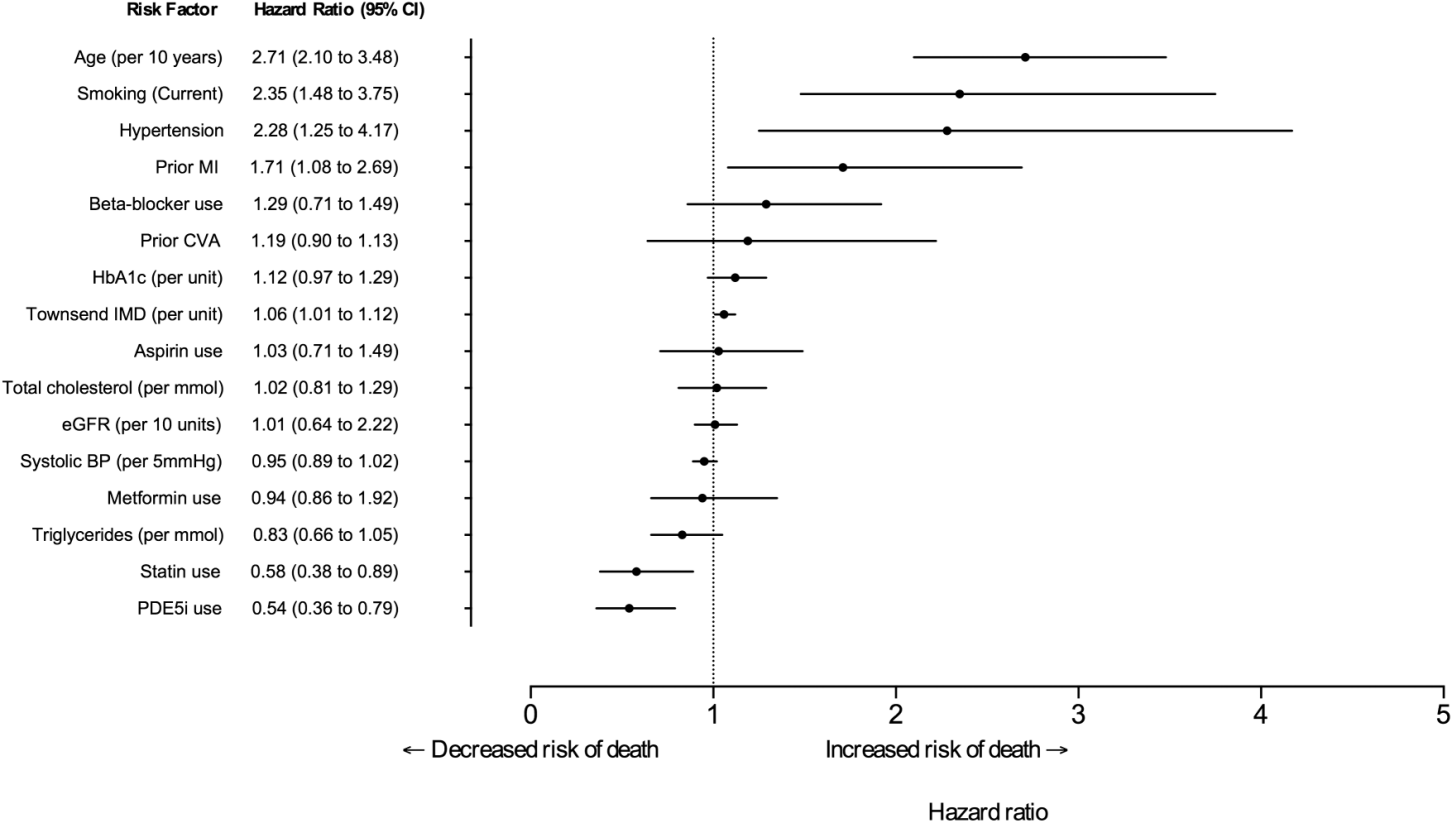
Forest plot of multivariable adjusted HRs of death associated with PDE5i use. PDE5i use adjusted for age, smoking, hypertension, prior MI, β-blocker use, prior CVA, HbA1c level, Townsend index of multiple deprivation (IMD) score, aspirin use, total cholesterol, estimated glomerular filtration rate, systolic BP, metformin use, triglyceride levels and statin use. BP, blood pressure; MI, myocardial infarction; PDE5i, phosphodiesterase type-5 inhibitor.

The lower risk of death in PDE5i users remained (HR=0.54 (0.36 to 0.80); p=0.002) after adjusting for age (per 10 years), eGFR (per 10 unit increase), smoking status (current or ex-smoker vs never), prior history of CVD, prior history of hypertension, known MI, systolic BP (per 5 mm Hg), use of a statin, metformin, aspirin and β-blockers, HDL cholesterol (per unit), total cholesterol (per unit), triglycerides (per unit), HbA1c (per unit), number of prescriptions and Townsend score (figure 2). Estimates from a competing-risk regression model were similar (see online supplementary figure S1).

#### Subgroup analyses

**Figure 3:**
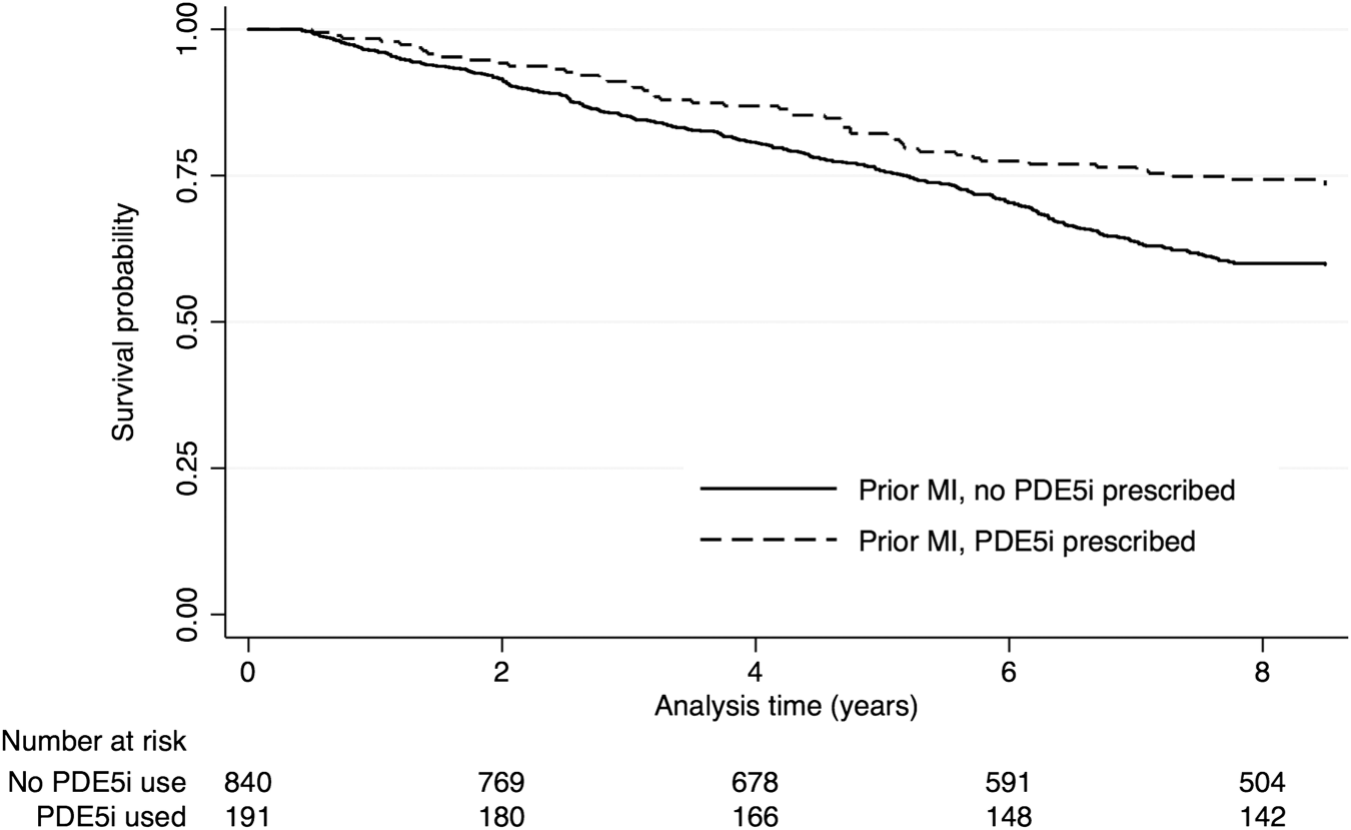
Time to all-cause mortality Kaplan-Meier curves for all men with a prior history of myocardial infarction (n=1031) by the treatment groups (PDE5i vs no PDE5i). PDE5i, phosphodiesterase type-5 inhibitor.

Among those with a prior history of MI (n=1031), there were fewer deaths in those previously treated with a PDE5i compared with those who were never treated (25.7% (49/191) vs 40.1% (337/840)). Figure 3 shows the Kaplan-Meier plot for men with a prior history of MI. The risk of mortality for those treated with a PDE5i was approximately 40% lower (HR=0.61 (0.45 to 0.81); p=0.001) in unadjusted models and by a quarter lower (0.73 (HR=0.53 to 0.99)) in models adjusted for age, smoking status and statin use (table 2).

**Table 2:** Adjusted HRs for mortality associated with PDE5i use by subgroups with prior history of co-morbid conditions in the naïve and imputed cohorts

	Naïve cohort unadjusted		Naïve cohort adjusted*		Imputed cohort*	
	HR (95% CI)	p Value	HR (95% CI)	p Value	HR (95% CI)	p Value
All men	0.69 (0.60 to 0.79)	<0.0001	0.83 (0.72 to 0.95)	0.009	0.73 (0.63 to 0.84)	<0.001
Prior AMI	0.59 (0.43 to 0.80)	0.001	0.73 (0.53 to 0.99)	0.045	0.63 (0.46 to 0.85)	0.003
Prior CCF	0.64 (0.47 to 0.87)	0.005	0.76 (0.55 to 1.04)	0.086	0.64 (0.46 to 0.87)	0.005
Prior AF	0.77 (0.58 to 1.02)	0.077	0.92 (0.68 to 1.25)	0.598	0.74 (0.55 to 0.99)	0.044
Prior stroke	0.65 (0.42 to 1.03)	0.068	0.82 (0.51 to 1.31)	0.045	0.62 (0.39 to 0.99)	0.045
Prior TIA	0.60 (0.38 to 0.95)	0.031	0.77 (0.47 to 1.24)	0.276	0.57 (0.36 to 0.92)	0.021
Prior PVD	0.66 (0.48 to 0.93)	0.016	0.76 (0.54 to 1.07)	0.123	0.69 (0.49 to 0.96)	0.030

Naïve cohort refers to the unimputed cohort.

*Adjusted for age, smoking status and statin use for each subgroup.

AF, atrial fibrillation; AMI, acute myocardial infarction; CCF, congestive cardiac failure; PDE5i, phosphodiesterase type-5 inhibitor; PVD, peripheral vascular disease; TIA, transient ischaemic attack.

In the other subgroups, there was an inverse association between PDE5i use and all-cause mortality. Those with a recorded history of congestive cardiac failure, TIA and PVD had 36%, 40% and 34% lower risk, respectively. The association in those with a prior history of atrial fibrillation or stroke was not significant. After adjustment for age, smoking status and statin use in multivariable analyses, the estimates for the association with mortality was attenuated (table 2). In a sensitivity analysis using an imputed cohort, the significant inverse association between PDE5i use and mortality, for each subgroup, remained after multivariable adjustment (table 2).

#### Effect of incident MI by PDE5i category

In the whole cohort, 432 men with no prior CVD history had an incident MI during the observation period. In this subgroup, the incidence of an AMI was less in those with a history of PDE5i use (64/1168, (5.2%). The IRR for an MI, derived from Poisson regression was 0.62 (0.49 to 0.80; p<0.0001) compared with those who were never prescribed PDE5i treatment (368/3757, (8.9%)). This difference remained after adjusting for age (8.5% vs 5.2%, p<0.0001; age-adjusted IRR 0.64 (0.50 to 0.83, p=0.001)).

**Figure 4:**
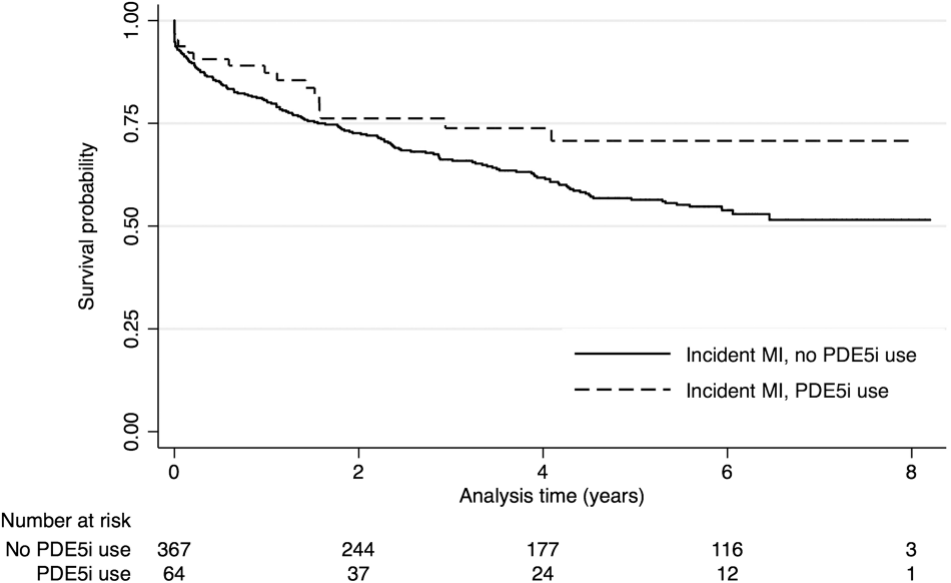
Time to all-cause mortality Kaplan-Meier curves for all men with an incident acute myocardial infarction (AMI) (n=432) by the treatment groups (PDE5i vs no PDE5i). Time at risk begins at date of incident AMI. PDE5i, phosphodiesterase type-5 inhibitor.

Mortality in PDE5i users with an incident MI during follow-up was also lower (25% vs 42.7%). In age-adjusted Weibull regression analyses, those prescribed a PDE5i had approximately a 40% lower mortality risk (HR=0.60 (0.54 to 0.69); p=0.001; figure 4). This lower mortality risk remained after adjusting for age, smoking status, hypertension, use of a statin, metformin, aspirin and β-blockers (HR=0.61 (0.53 to 0.69), p=0.001). In sensitivity analyses, estimates from a similarly adjusted competing-risk model indicated that mortality in PDE5i users were around 50% lower (HR 0.52 (0.44 to 0.60). p=0.001.

## Discussion

ED is strongly associated with the onset of ischaemic heart disease, with a reported average of 3 years between onset of ED and subsequent cardiovascular events,^26^ representing an important window for aggressive risk factor reduction. In patients with type 2 diabetes mellitus and ED at high cardiovascular risk, further adjunctive therapies are necessary to protect against the onset of future cardiovascular events.

The major findings of our observational study of 5956 men with type 2 diabetes mellitus followed for a median period of 7 years are threefold. First, PDE5i use was associated with a significantly lower all-cause mortality rate and a lower proportion of deaths. This lower mortality risk in those taking a PDE5i persisted after adjustment for known risk modifiers including previous stroke, previous AMI, age, eGFR, PVD, hypertension and use of cardioprotective agents such as β-blockers and statins. Second, patients taking a PDE5i had significantly lower incidence of AMI over the study period. Third, in a subgroup analysis of patients with history of AMI or an incident AMI during the study period, PDE5i use was associated with significantly lower mortality risk. Thus PDE5i use was associated with (1) lower frequency of AMI and (2) lower mortality in patients with AMI, both of which contribute to lower overall mortality.

### Comparison with other studies and possible mechanisms

A previous US-based study in patients with diabetes having silent angiographically proven coronary artery disease showed an association between PDE5i use and lower risk of major adverse cardiac events in univariate analyses, suggesting that PDE5i use might lower the risk in patients with diabetes having coronary artery disease and ED. However, in multivariate analyses PDE5i use did not attain statistical significance likely due to the relatively small sample size (n=291).^9^

It is well recognised that 3′,5′-cyclic guanosine monophosphate (cGMP) and its central target protein kinase G (PKG) attenuate stress responses in the heart, and there is compelling evidence that activation of this pathway serves to reduce pathological hypertrophy, to protect against IRI, to dampen cardiac responses to catecholamines and to enhance cell survival and mitochondrial function.^27^ The PDE5 enzyme catalyses the hydrolytic breakdown of cGMP and is constitutively expressed in cardiac muscle in addition to the vasculature. The PDE5is sildenafil, tadalafil and vardenafil act to increase cellular cGMP by preventing its hydrolysis by PDE5. In animal models of myocardial ischaemia and IRI, PDE5i use has reduced infarct size, improved left ventricular performance, suppressed ischaemia-induced arrhythmias and improved animal survival.^12^ ^13^ ^15^ ^28–30^ A number of proposed mechanisms for their cardioprotective actions in IRI exist including activation of PKG through the nitric oxide signalling pathway,^31^ ^32^ opening of mitochondrial ATP-sensitive potassium channels^28^ and stimulation of the sarcolemmal Na+−K+ ATPase pump.^33^ In addition to direct myocardial effects, PDE5is may afford protection against endothelial reperfusion injury,^34^ improve vascular endothelial function and reduce systemic and pulmonary BP.^35^ ^36^

ED is a strong predictor of subsequent ischaemic heart disease, particularly at younger ages and in intermediate-risk groups.^37^ It may appear paradoxical therefore that PDE5i use in our study was associated with reduced mortality. However, in addition to the aforementioned animal studies of PDE5 inhibition in AMI, clinical trials of PDE5i use in heart failure have demonstrated improved parameters of cardiac performance^38^ and quality of life indices.^16^ ^39^ Similarly, in a randomised trial, chronic therapy with tadalafil in men with increased cardiovascular risk improved endothelial function as assessed by flow-mediated dilatation and plasma endothelial-1 levels.^40^ It is therefore possible that in this cohort of patients with high prevalence of pre-existing CVD, PDE5is exert protective effects large enough to offset the increased cardiovascular risk associated with ED. The reported association between PDE5i use and reduced mortality, 70% of which is estimated to be secondary to cardiovascular causes,^41^ ^42^ does not provide any clues as to the underlying mechanism of protection. From the data available in animal studies, arterial vasodilatation, reduction in myocardial infarct size and thus reduced progression to ischaemic heart failure are likely contributors. In addition, early in vivo data in animal models suggest that PDE5i has antiarrhythmic effects during ischaemia and post-MI.^29^ ^43^

### Strengths and limitations

The study design does carry some limitations. There are inherent selection bias issues associated with inferring treatment effects using observational data. The observational nature of the design does not establish causality and well-conducted RCTs have the advantage over observational studies by controlling for both known and unknown or unmeasured confounding factors (eg, life course socioeconomic position and doctor selection practices). It is plausible that men treated with PDE5is were at lower risk due to younger age and existence of other residual confounding factors that we were unable to ascertain and control. We did not have reliable information concerning patient activity levels, partner status or frequency of sexual intercourse, all of which are associated with improved life expectancy.^44–46^ Furthermore, data on incidence and prevalence of ED were not available in this patient cohort and we were therefore unable to account for this in multivariate analysis. We were however able to adjust for a number of intergroup differences using several methods including propensity-matching and multiple imputations to provide robust findings. Furthermore, the study has a number of strengths including its size and the duration of follow-up in a demographically stable primary care setting.

## Conclusion

Our longitudinal data in a primary care setting demonstrate that PDE5i use is associated with lower mortality in men with type 2 diabetes at high risk of CVD. RCTs are called for to determine whether chronic PDE5 inhibition improves outcomes in patients with CVD and/or type 2 diabetes. Further evidence is also required to elucidate the mechanism of PDE5i-associated cardioprotection. The strong relationship between PDE5i use and lower mortality in type 2 diabetes requires urgent investigation because there could be considerable clinical benefit beyond the treatment of ED.

**Key messages**

**What is already known on this subject?**

- Evidence from animal models of acute myocardial infarction has indicated potential cardioprotective actions of phosphodiesterase type-5 (PDE5) inhibition, including reduction in infarct size, improvement in contractile function and suppression of cardiac dysrhythmias. In addition, small-scale randomised trials of PDE5 inhibitors in systolic heart failure have shown improvements in patient haemodynamic parameters, functional indices and good tolerability and safety. There are currently no available long-term data concerning the effect of PDE5 inhibition on survival in patients who are at high risk of cardiovascular disease.

**What might this study add?**

- We undertook a retrospective analysis of mortality in a cohort of patients with type 2 diabetes mellitus (T2DM) and therefore high-attendant cardiovascular risk. Our findings demonstrated that, for the first time, PDE5 inhibitor use is associated with significantly reduced mortality in patients with T2DM, an effect which remained after multiple adjustments for known confounding factors.

**How might this impact on clinical practice?**

- Our findings provide strong evidence for PDE5 inhibitors acting to reduce mortality in T2DM. Further evidence is required to elucidate the role of PDE5is in cardioprotection.
