# Supplementary material for: Phosphodiesterase type-5 inhibitor use in type 2 diabetes is associated with a reduction in all-cause mortality
Source: Heart. 2016 Aug 1;102(21):1750–6. doi: 10.1136/heartjnl-2015-309223 (PMC5099221; doi:10.1136/heartjnl-2015-309223)

Supplementary Appendix

Phosphodiesterase type-5 inhibitor use in type 2 diabetes is associated with a reduction in all cause mortality

Running title: *Phosphodiesterase type-5 inhibitor use in diabetes and mortality*

Simon G Anderson[1, 2†], *NIHR Clinical Lecturer in Cardiology*
David C Hutchings[1†], *BHF Clinical Research Training Fellow and Cardiology SpR*
Mark Woodward [2,3], *Professor of Statistics and Epidemiology*
Kazem Rahimi[2], *Professor and James Martin Senior Fellow in Essential Healthcare*
Martin K Rutter[4,5], *Consultant in Endocrinology*
Mike Kirby[6], *Professor of Primary Care Medicine*
Geoff Hackett[7], *Professor of Men's Health and Consultant in Urology*
Andrew W Trafford[1], *Professor of Cardiac Pathophysiology*
Adrian H Heald [8,9], *Consultant in Endocrinology and Research Fellow, Manchester University*

[†]Joint first authorship

[1]Institute of Cardiovascular Sciences, University of Manchester;

[2]The George Institute for Global Health, Oxford Martin School, University of Oxford, 34 Broad Street, Oxford OX1 3BD, United Kingdom;

[3]The George Institute for Global Health, University of Sydney, Australia;

[4]Manchester Diabetes Centre, Manchester, Central Manchester University Hospitals NHS Foundation Trust, UK

[5]Centre for Endocrinology and Diabetes, Institute of Human Development, University of Manchester, UK

[6]The Centre for Research in Primary & Community Care, University of Hertfordshire;

[7]Department of Urology, Good Hope Hospital, Sutton Coldfield;

[8]School of Medicine, and Manchester Academic Health Sciences Centre, University of Manchester;

[9]Leighton Hospital, Crewe, United Kingdom

Supplement Table 1. The anthropometric and clinical characteristics from Imputed and Propensity analyses.

	Imputed		Propensity Matched	
	Not prescribed a PDE5i	Prescribed a PDE5i	Not prescribed a PDE5i	Prescribed a PDE5i
N (%)	4,597 (77.2)	1,359 (22.8)	513 (82.6)	108 (17.4)
Median number of prescriptions[a]		16 (2-173)		17 (7-56)
Age (years)	72.8 (72.4 - 73.1)	71.2 (70.7 - 71.6)**	74.3 (73.4 – 75.1)	72.8 (71.3 – 74.4)
HbA1c (%)	7.3 (7.2 - 7.3)	7.4 (7.3 - 7.5)*	7.3 (7.2 - 7.4)	7.5 (7.2 - 7.8)
BMI kg/m^2	30.3 (30.1 - 30.5)	30.6 (30.2 - 30.9)	30.2 (29.6 - 30.8)	30.9 (29.7 – 32.1)
LDL (mmol/l)	2.17 (2.14 - 2.19)	2.20 (2.15 - 2.24)	2.12 (2.05 – 2.19)	2.21 (2.07 – 2.35)
HDL (mmol/l)	1.15 (1.13 – 1.16)	1.15 (1.13 – 1.17)	1.11 (1.08 – 1.14)	1.13 (1.07 – 1.19)
Cholesterol (mmol/l)	4.08 (4.05 - 4.11)	4.10 (4.05 - 4.17)	4.02 (3.94 – 4.10)	4.08 (3.91 – 4.24)
Albumin g/L	41.6 (41.5 - 41.7)	41.5 (41.3 - 41.7)	41.3 (41.0 – 41.6)	41.2 (40.5 – 41.8)
Creatinine micromole/L	104 (102 - 105)	100 (98 - 102)*	111 (107 – 114)	102 (98 – 107)
eGFR (MDRD)	68 (67 - 69)	70 (69 - 71)**	64 (62 – 65)	68 (65 - 77)*
Systolic BP (mmHg)	138 (137 - 138)	138 (137 - 139)	136 (135 – 138)	138 (135 – 141)
Diastolic BP (mmHg)	77 (76 - 77)	77 (76 - 77)	74 (73 - 75)	76 (75 – 78)*
Smoking status				
Never smoked	1,375 (30.0)	418 (30.7)	98 (19.1)	32 (29.6)
Ex-smoker	2,109 (45.9)	637 (46.9)	263 (51.3)	53 (49.1)
Current smoker	1,113 (24.2)	304 (22.4)	152 (29.6)	23 (21.3)
Any statin use	2,997(65.2)	1,092 (80.4)**	427 (83.2)	95 (87.9)
Clopidogrel use	237 (5.2)	55 (4.0)	53 (10.3)	14 (13.0)
Aspirin use	1,894 (41.2)	714 (52.5)**	360 (70.2)	71 (65.7)
Metformin use	2,126 (46.2)	874 (64.3)**	278 (54.2)	77 (71.3)**

Beta-blocker use	1,303 (28.3)	338 (24.9)*	263 (51.3)	47 (43.5)
History of prior MI	840 (18.3)	191 (14.0)**	276 (53.8)	52 (48.1)
Known Atrial Fibrillation	680 (14.8)	165 (12.1)*	115 (22.4)	19 (17.6)
Known Hypertension	3,062 (66.6)	963 (70.8)**	355 (69.2)	83 (76.9)
Peripheral vascular disease	518 (11.3)	145 (10.7)	93 (18.1)	20 (18.5)
Stroke	367 (8.0)	68 (5.0)**	48 (9.4)	7 (6.5)
TIA	325 (7.1)	78 (5.7)	61 (11.9)	12 (11.1)
Congestive Cardiac Failure	511 (11.1)	108 (8.0)**	160 (31.2)	24 (22.2)

Data imputed from most recent measure within 1 year of study entry; **P<0.001; *P<0.05. [a]Median and interquartile range

Supplementary Figure Legend

1. Supplement Figure 1. Forest plot of multivariable adjusted hazard ratios of death associated with PDE5i use from a competing risk regression model.

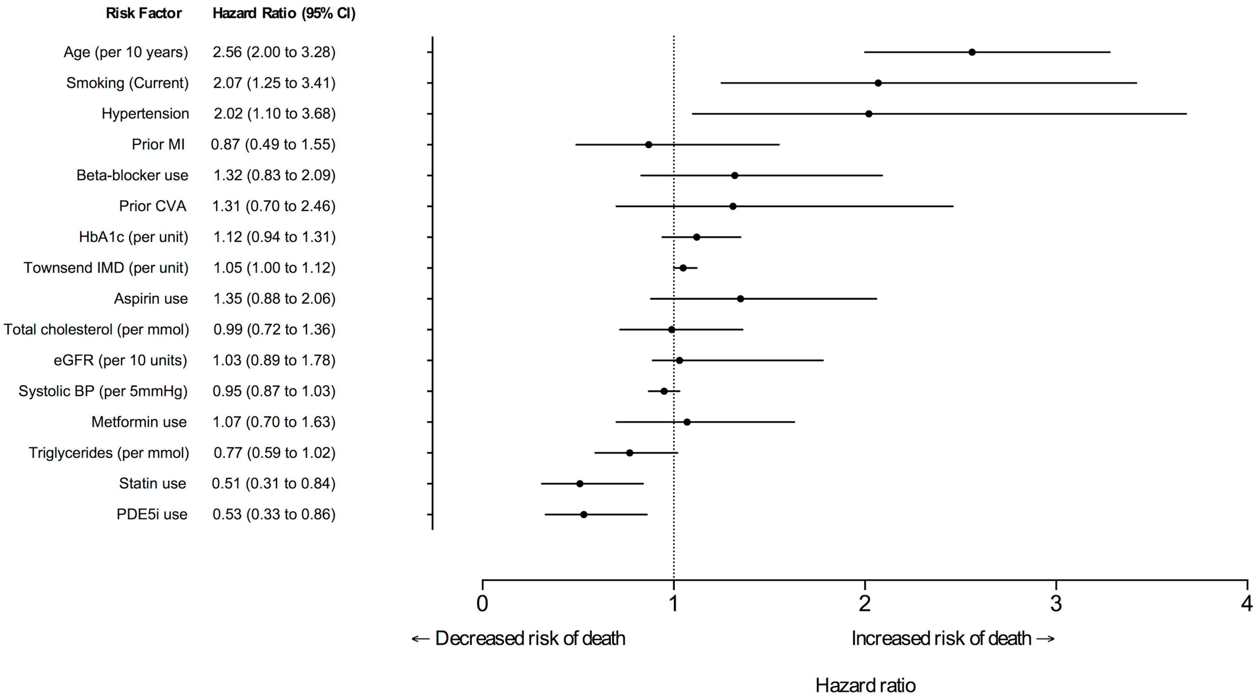

Supplement: Supplementary appendix [file heartjnl-2015-309223supp_appendix.pdf]
